# Mirtazapine: A One-Stop Strategy for Treatment of Opioid Withdrawal Symptoms

**DOI:** 10.7759/cureus.43821

**Published:** 2023-08-20

**Authors:** Elisha Lalani, Raakhi Menon, Mariam A Mufti, Cecil Kumfa, Mukaila Raji

**Affiliations:** 1 Department of Internal Medicine, Division of General Medicine, University of Texas Medical Branch, Galveston, USA; 2 Department of Internal Medicine, Division of Geriatrics & Palliative Medicine, University of Texas Medical Branch, Galveston, USA

**Keywords:** depression, polypharmacy, withdrawal, opioid, mirtazapine

## Abstract

Public health efforts to reduce the opioid overdose epidemic and treat opioid use disorder (OUD) have met with challenges associated with current non-standardized approaches to managing opioid withdrawal symptoms, such as itching, jitteriness, anxiety, depression, craving, vomiting, diarrhea, insomnia, and anorexia. These symptoms pose substantial obstacles to the safe initiation of medications for OUD, maintenance of long-term sobriety, and prevention of relapse. In clinical practice, multiple medications (polypharmacy) are prescribed to manage these withdrawal symptoms, including ondansetron and promethazine for vomiting and nausea, loperamide and Lomotil for diarrhea, hydroxyzine and doxepin for pruritus, benzodiazepines, the Z-drugs, and melatonin for insomnia, and benzos, tricyclic antidepressants (TCAs), and various serotonergic agents for anxiety. This polypharmacy is associated with an increased risk of adverse drug-drug interactions and adverse drug events, increased medical costs, and increased odds of medication non-adherence and relapse. We propose an alternative single medication, mirtazapine, a noradrenergic and specific serotonergic receptor antagonist, that can be used for myriad symptoms of opioid withdrawal. Case series, clinical studies, and clinical trials have shown mirtazapine to be effective for treating nausea and vomiting resulting from multiple etiologies, including hyperemesis gravidarum and chemotherapy-induced emesis. Other evidence supports the salutary effects of mirtazapine on itching and craving. Research findings support mirtazapine’s beneficial effects on diarrhea and anxiety, a consequence of its modulating effects on serotonergic receptors mediating mood and gastrointestinal symptoms. There is also evidence supporting its efficacy as a potent and non-addictive sleep aid, which presents itself as a solution for insomnia associated with opioid withdrawal. The current review presents evidence from extant literature supporting mirtazapine as a one-drug strategy to treat the variety of symptoms of opioid withdrawal. This one-drug strategy has much potential to decrease polypharmacy, adverse drug events, relapse, and healthcare cost and increase the likelihood of prolonged sobriety and better quality of life for people living with OUD.

## Introduction and background

The United States Department of Health and Human Services (DHHS) declared the opioid overdose epidemic a public health emergency in 2017 [1]. Various strategies have since been proposed and undertaken to combat the opioid crisis, but implementing the treatment of opioid use disorder (OUD) faces significant challenges due, in part, to the difficulty of effectively managing the distressful symptoms of opioid withdrawal, including itching, craving, vomiting, nausea, diarrhea, and insomnia. Suboptimal management of opioid withdrawal symptoms poses a substantial challenge to initiating OUD treatment and preventing relapse. In real-world practice, myriad medications in different pharmacologic categories are prescribed to manage each individual withdrawal symptom such as specific agents for vomiting and nausea (ondansetron), diarrhea (loperamide), pruritus (hydroxyzine), insomnia (benzodiazepines, Z-drugs), and anxiety (benzodiazepines, selective serotonin reuptake inhibitors (SSRIs)). This practice often leads to polypharmacy and creates a high potential for drug-drug interactions, increased medical costs, and increased burden of unintended side effects, all of which increase the risk of relapse and decrease the odds of initiating treatment for OUD and maintaining sobriety. 

OUD modifies serotonergic and dopaminergic pathways via the nucleus accumbens connections to limbic and autonomic brain regions, which contributes to the development of addictive behaviors and withdrawal symptoms across multiple organ systems [2]. Opioid withdrawal is fraught with many challenges mostly resulting from noradrenergic hyperactivity, with early symptoms including anxiety, agitation, aches, insomnia, sweating, yawning, and runny nose [3]. These symptoms can be severely distressing and precipitate relapse and continued opioid dependency. Mirtazapine is a potent antagonist of serotonin (5-HT2 and 5-HT3) receptors, a strong antihistamine, and an antagonist for central alpha-adrenergic autoreceptors and heteroreceptors. Consequently, mirtazapine is sometimes described as a noradrenergic and specific serotonergic antidepressant (NaSSA) [3].

We propose a single drug, mirtazapine, as a one-drug strategy to treat symptoms of opioid withdrawal. With its multiple pleiotropic effects in the enteric and central nervous systems, mirtazapine has clinical and basic science evidence to support its use for treating many symptoms of opioid withdrawal, thereby reducing polypharmacy, total medication cost, drug-drug interactions, and the burden of unintended side effects while improving adherence to OUD treatment. In this review, we highlight evidence from the extant literature to demonstrate how mirtazapine ameliorates each symptom of opioid withdrawal.

## Review

Evidence for mirtazapine as a one-drug therapy for myriad opioid withdrawal symptoms

Nausea and Vomiting

One of the most common symptoms of opioid withdrawal is nausea and/or vomiting. In the enteric and central nervous systems, 5-HT receptors are prevalent and have been known for decades to participate in emetic activity. The subtype 5-HT3 receptor antagonists are often used to prevent nausea and vomiting due to secondary causes, such as chemotherapy, hyperemesis gravidarum, and radiation-induced and postoperative nausea [4,5]. Specifically, mirtazapine poses antagonistic effects on the 5-HT3 receptor, giving the drug its antiemetic properties [6]. Mirtazapine was shown to substantially reduce nausea and vomiting in patients, including those who had unsuccessful courses with other antiemetic drugs in case reports, cohort studies, and RCTs [6,7,4] In the study by Malamood et al., mirtazapine significantly decreased nausea and vomiting in patients with refractory gastroparesis at two and four weeks, respectively [7]. A similar effect has been seen in cancer patients receiving chemotherapy. Chemotherapy drugs, such as cisplatin, activate the 5-HT3 receptor [8], whereas mirtazapine directly antagonizes this receptor and improves nausea and vomiting caused by chemotherapy. Mirtazapine has also been shown to be an effective medication for non-mechanical vomiting after gastric bypass [9]. Much evidence supports mirtazapine as an effective therapy to treat nausea and vomiting of various etiologies. We, therefore, argue for mirtazapine as a potential treatment for nausea and vomiting, which are common and highly distressing symptoms of opioid withdrawal.

Diarrhea

Another common and debilitating symptom of opioid withdrawal is diarrhea, which is mediated, at least in part, by the 5-HT3 receptor. Loperamide, yet another medication to treat opioid withdrawal symptoms, is typically prescribed for symptomatic management; however, mirtazapine has been shown in randomized controlled trials (RCTs) to also relieve this symptom. Many patients with chronic diarrhea are deemed to have irritable bowel syndrome, diarrhea type (IBS-D). A groundbreaking double-blind RTC tested the effectiveness of mirtazapine in controlling diarrhea and found that mirtazapine significantly improves diarrhea in patients with IBS-D [10]. 

Mirtazapine has been demonstrated to be efficient in improving diarrhea, especially in cases with comorbid depression or anxiety [11]. Diarrhea is found to be worse when patients also have mood disorders. Lu et al. recruited patients meeting the Rome III criteria for IBS-D; 118 of the 410 participants in their study had comorbid depression [12]. Patients with concomitant depression did not experience relief of abdominal pain after defecation. These patients also had a higher score of anxiety and somatization due to the passing of mucus and overlapping functional dyspepsia. About one-third of patients were prescribed neuromodulators, and mirtazapine showed improvement in abdominal pain, discomfort, and diarrhea in these patients [12]. Extensive research already exists showing a correlation between IBS and mood disorders, such as depression and anxiety [12,13]. These studies support the use of a single medication, such as mirtazapine, that can treat both mood disorders and co-occurring diarrhea [10,12]. This reflects, at least in part, the shared serotonergic pathways via the same 5HT receptors, which mirtazapine blocks, thereby explaining the anti-depressant, anti-anxiety, and anti-diarrhea effects of the drug.

Anxiety, Jitteriness, Jumpiness, and Depression

Through its myriad influences on multiple serotonergic and noradrenergic receptors, mirtazapine has demonstrated promise in decreasing the severity of subjective opioid withdrawal symptoms, such as agitation and anxiety [14]. This decrease is thought to be mediated by an increase in dopamine levels, with some relief of these symptoms by mirtazapine blockade of the 5-HT2c receptors in the prefrontal cortex and of alpha-2 adrenoceptors in the cerebral cortex [15]. 

In a pilot study of Alzheimer's disease patients, a mean of 23 mg/day of mirtazapine resulted in a significant reduction in agitation [16]. Another study, however, observed no benefit of mirtazapine compared with a placebo, plus potentially higher mortality, and therefore discouraged the use of this drug for treating anxiety in Alzheimer's disease patients [17]. It is thus important to fully evaluate the potential of this drug for the treatment of anxiety and jitteriness in patients experiencing opioid withdrawal symptoms. These mixed findings underscore the need for more rigorous comparative effectiveness and toxicity studies of other CNS-acting drugs (e.g. serotonergic agents, anti-epileptic drugs) as potentially safer alternatives to antipsychotics (commonly used for psychomotor agitation) in the setting of agitated behaviors occurring in OUD patients experiencing severe withdrawal symptoms.

Nevertheless, mirtazapine has been shown in many clinical trials to significantly help with depression and is widely accepted as an effective FDA-approved antidepressant [18]. For example, in a four-week, prospective, open-labeled study of cancer patients, mirtazapine significantly improved depression within one week [19]. In addition to depression, mirtazapine also reduced co-occurring nausea, vomiting, and insomnia. 

In managing jitteriness in opioid withdrawal settings, evidence shows that among all antidepressants and anxiolytics, mirtazapine not only has the lowest odds of jitteriness but may also reduce these symptoms [20,21]. Jitteriness is a symptom that falls under the umbrella term of anxiety. Jitteriness frequently coexists with depression and often occurs in opioid withdrawal settings. Medications with SSRIs and serotonin and norepinephrine reuptake inhibitors (SNRIs) can cause a transient increase in anxiety symptoms, including jitteriness. Mirtazapine has been linked to an early relief of these anxiety-provoked symptoms. Another study also shows mirtazapine is superior to placebo in patients with depression and anxiety [21]. Thus, using mirtazapine as the first-line treatment for opioid withdrawal symptoms of anxiety, with its potential palliation of jitteriness/jumpiness, reduces polypharmacy.

Insomnia

Insomnia is a classic symptom of opioid withdrawal, persisting even in patients undergoing tapering and detoxification programs. Apart from its expected impact on quality of life, insomnia can also enhance drop-out, relapse, and suicide rates, ultimately hampering the success of OUD treatment efforts. Unfortunately, current medications for the management of insomnia in these patients such as benzodiazepines and tricyclic antidepressants are frequently toxic or addictive, necessitating the search for better alternatives [22]. Though mirtazapine is frequently used as an antidepressant, there is evidence of its effectiveness in the treatment of symptoms of opiate withdrawal, specifically insomnia, co-occurring anxiety, anorexia, nausea, and vomiting [23]. In particular, the antihistamine effect on H1 receptors partly mediates the sedation and appetite-inducing effects of mirtazapine, which benefits older patients who may be frail and have sleep disturbances and poor appetite when depressed. We suggest that these effects could also benefit opioid withdrawal-associated symptoms of insomnia and appetite loss.

A review of 23 clinical trials for the sedative effects of mirtazapine in patients with major depressive disorder demonstrated significant improvement in sleep efficiency, quality, and total time [24]. In a more recent study, mirtazapine was dosed at 7.5 mg as part of a randomized, double-blinded, cross-over placebo-controlled trial to evaluate its effects on normal and disturbed sleep in 19 healthy men. Next-day alertness and cognitive function were also assessed. Total sleep time increased by half an hour, the number of awakenings reduced by 35-40%, and daytime sleepiness increased while sustained attention declined, confirming that mirtazapine at low doses could be used for the treatment of insomnia [25]. 

Furthermore, another study showed increased total sleep time in cancer patients treated with mirtazapine. About one-third of all patients experienced an increase in sleepiness, but it gradually lessened over a few days [19]. In general, many studies have demonstrated that mirtazapine increases sleepiness, helps with insomnia, and is often used clinically for insomnia, especially in older patients, due to a relatively safer side effect profile than many other pharmaceuticals available for insomnia.

Anorexia and Low Appetite

Additionally, weight gain, a potentially adverse effect associated with mirtazapine, can be used to overcome anorexia and appetite suppression often linked to opioid withdrawal. In both long-term (>4 months) and short-term (4-12 weeks) use, there is an average of 1.74-2.59 kg weight gain, respectively [26]. The main mechanism by which this medication restores appetite is unknown, though studies have found an acceleration in gastric emptying, possibly due to 5-HT2C antagonism/inverse agonism [27]. The antihistaminic effects of mirtazapine could also contribute to the increase in appetite. 

Of the few pharmacological treatment options available for cancer-related anorexia, mirtazapine remains a strong choice. An open-label single-institution phase II trial of mirtazapine was conducted with non-depressed patients with cachexia and anorexia related to underlying cancer. The study found that 24% of patients gained at least 1 kg after four weeks of therapy [28]. Cachexia secondary to cancer can present in a variety of ways due to different mechanisms. For example, pro-inflammatory cytokine activity, which leads to muscle wasting and entero-hormonal production, also leads to early satiety and anorexia [29]. In this study, patients reported a decrease in fatigue, nausea, and early satiety; reducing these symptoms corresponded with weight gain. In sum, evidence from other settings supports the consideration of prescribing mirtazapine for opioid withdrawal-related anorexia and weight loss.

Itching

Of the opioid withdrawal symptoms, itching symptoms stand out as a major contributor to psychological and physical distress, which heightens the risk of relapse. Mirtazapine has been used in other clinical conditions for itching [30-36]. These conditions include liver failure, renal failure, uremic cholestasis, psoriasis, atopic dermatitis, primary and metastatic cancers, lymphomas and leukemias, and cholestasis [31]. Other studies presented evidence of the effectiveness of mirtazapine in managing itching in patients on hemodialysis [32] and patients with morphine-induced itching [34]. Studies on the role of mirtazapine in severe pruritus suggest it as a potential intervention to reduce pruritus in patients experiencing symptoms of opioid withdrawal [31-36]. These findings also underscore the need for RCTs for the effectiveness of mirtazapine as a potential treatment for itching and other distressing symptoms during opioid withdrawal.

Tremors

Tremors, another distressing symptom of opioid withdrawal, may also respond to mirtazapine, as evidenced by findings from an open-label, observer-blind study that evaluated the clinical efficacy of untreated essential tremors in a group of 30 patients. Of these, 85% of patients who received mirtazapine experienced a statistically significant benefit in improvement of tremors. At one year, 55% continued to have control of their tremors compared to 50% reporting limited lasting benefits with commonly used propranolol and primidone [37]. 

A case series documented improvement or resolution of Parkinsonian tremors, action tremors (postural and kinetic), and levodopa-induced dyskinesias in five patients taking 30 mg of mirtazapine. A return of symptoms was observed in two patients upon cessation and with a resolution of tremors upon restarting [38].

These findings demonstrate that mirtazapine may be as effective as, if not better than, commonly used medications for various types of tremors. But it is important to note that the mechanism behind how mirtazapine may facilitate the control of opiate withdrawal tremors is unknown and that further studies are warranted.

Cravings

Evidence of mirtazapine’s role in mitigating cravings in patients with other substance use disorders has also been well documented [14,39-44]. Evidence from case reports, case series, and RCTs shows some effectiveness of mirtazapine in the management of cravings and relapse in patients with addiction to cocaine, methamphetamine, and alcohol. Given the shared mechanisms of addictive substances (vis-à-vis-their effect on dopamine pathways in the nucleus accumbens), mirtazapine has the potential to reduce cravings and mitigate relapse in patients with OUD. There is an ongoing clinical trial of mirtazapine as a treatment of OUD and amphetamine-type stimulants in patients already on Mainstreaming Addiction Treatment (MAT)-buprenorphine maintenance [43]. A larger RCT is key to further defining the roles of mirtazapine as a potential intervention in patients with OUD experiencing withdrawal symptoms.

Summary of evidence from basic science and clinical data

Mirtazapine exerts its effects on many different types of receptors: antagonist of serotonin (5-HT2 and 5-HT3), histamine (H1), and noradrenergic receptors (α2). Several case series, clinical studies, and clinical trials have demonstrated the positive effects of mirtazapine on multiple opioid withdrawal symptoms. Mirtazapine causes indirect enhancement of 5-HT1 receptors, which improves mood and treats depression and anxiety. By blocking 5-HT2 and 5-HT3 receptors, mirtazapine prevents nausea and vomiting. RCTs have shown mirtazapine to provide significant improvement in diarrhea. The antihistaminic effects of mirtazapine cause decreased pruritus, insomnia, and anorexia. Limited studies and case reports have shown improvement in tremors; however, the mechanism of action is still unclear, warranting further research. RCTs show some effectiveness of mirtazapine in the management of cravings and relapse in cocaine use disorder. This can be translated to OUD, as drug addiction and cravings employ the same noradrenergic pathway.

Clinical implications

Mirtazapine has been used in a variety of clinical scenarios to help with symptoms (stemming from multiple etiologies) of nausea, vomiting, diarrhea, depression, insomnia, anorexia and low appetite, itching, tremors, and cravings and addiction. Basic science and clinical data have been presented on how these symptoms occur and how mirtazapine prevents them. Through clinical data, it is clear that mirtazapine has the potential to address multiple symptoms of opioid withdrawal, a one-drug strategy that is safer than the current approach of using one drug for each symptom [44-46]. Most drugs currently used for the treatment of opioid dependency may limit aspects of withdrawal symptoms, but their availability might also be limited, as some of these drugs (e.g. benzodiazepines) are controlled substances. Concomitant use of non-scheduled medications is therefore encouraged, especially those that could have combined effects on dependency and withdrawal symptoms. Another medication, lofexidine hydrochloride, is currently approved by the FDA for opioid withdrawal syndrome [46,47]. This is an alpha-2 adrenergic agonist that works by decreasing sympathetic tone and preventing norepinephrine release [44]. This medication can only be used for two weeks, but withdrawal symptoms often persist much longer than two weeks. Lofexidine hydrochloride can temporarily help with cravings and may treat immediate life-threatening issues, such as hypertension or hyperthermia, but unlike mirtazapine, it does not help with other distressing symptoms of withdrawal, as described in this review.

None of the current strategies use a single medication to control all, or even most, of the symptoms of opioid withdrawal. Multiple medications are typically used, posing an additional challenge that patients must face to overcome OUD. Mirtazapine has much potential in the OUD setting as a one-drug approach to minimize polypharmacy, reduce risks of drug-drug and drug-disease interactions, and reduce cost while improving adherence to medications and lifestyle interventions to maintain long-term sobriety. In this review, we used evidence of mirtazapine helping symptoms stemming from etiologies other than OUD or withdrawal. We advocate for a large RCT aimed at examining the efficacy and effectiveness of mirtazapine in treating opioid withdrawal symptoms. 

## Conclusions

Mirtazapine alleviates multiple symptoms of opioid withdrawal and thus has potential to reduce polypharmacy and adverse drug events. Mirtazapine, a one-drug strategy for opioid withdrawal symptoms, can hasten initiation of critical medications for OUD. Its impact on polypharmacy can lessen healthcare costs and improve adherence to lifestyle interventions to maintain sobriety. Mirtazapine’s salutary effects on opioid withdrawal symptoms merit a large randomized, controlled, comparative effectiveness trial. 
